# Adaptive mutations in sugar metabolism restore growth on glucose in a pyruvate decarboxylase negative yeast strain

**DOI:** 10.1186/s12934-015-0305-6

**Published:** 2015-08-08

**Authors:** Yiming Zhang, Guodong Liu, Martin K M Engqvist, Anastasia Krivoruchko, Björn M Hallström, Yun Chen, Verena Siewers, Jens Nielsen

**Affiliations:** Department of Biology and Biological Engineering, Chalmers University of Technology, Kemivägen 10, 412 96 Göteborg, Sweden; Science for Life Laboratory, KTH-Royal Institute of Technology, 171 21 Stockholm, Sweden; Novo Nordisk Foundation Center for Biosustainability, Technical University of Denmark, Kogle Allé 6, 2970 Hørsholm, Denmark; Novo Nordisk Foundation Center for Biosustainability, Chalmers University of Technology, Kemivägen 10, 412 96 Göteborg, Sweden

**Keywords:** Pyruvate decarboxylase, Genomic DNA sequencing, Yeast, Reverse engineering, *MTH1*, Hexose transporter, Citrate synthase, Histone deacetylase

## Abstract

**Background:**

A *Saccharomyces cerevisiae* strain carrying deletions in all three pyruvate decarboxylase (PDC) genes (also called Pdc negative yeast) represents a non-ethanol producing platform strain for the production of pyruvate derived biochemicals. However, it cannot grow on glucose as the sole carbon source, and requires supplementation of C_2_ compounds to the medium in order to meet the requirement for cytosolic acetyl-CoA for biosynthesis of fatty acids and ergosterol.

**Results:**

In this study, a Pdc negative strain was adaptively evolved for improved growth in glucose medium via serial transfer, resulting in three independently evolved strains, which were able to grow in minimal medium containing glucose as the sole carbon source at the maximum specific rates of 0.138, 0.148, 0.141 h^−1^, respectively. Several genetic changes were identified in the evolved Pdc negative strains by genomic DNA sequencing. Among these genetic changes, 4 genes were found to carry point mutations in at least two of the evolved strains: *MTH1* encoding a negative regulator of the glucose-sensing signal transduction pathway, *HXT2* encoding a hexose transporter, *CIT1* encoding a mitochondrial citrate synthase, and *RPD3* encoding a histone deacetylase. Reverse engineering of the non-evolved Pdc negative strain through introduction of the *MTH1*^*81D*^ allele restored its growth on glucose at a maximum specific rate of 0.053 h^−1^ in minimal medium with 2% glucose, and the *CIT1* deletion in the reverse engineered strain further increased the maximum specific growth rate to 0.069 h^−1^.

**Conclusions:**

In this study, possible evolving mechanisms of Pdc negative strains on glucose were investigated by genome sequencing and reverse engineering. The non-synonymous mutations in *MTH1* alleviated the glucose repression by repressing expression of several hexose transporter genes. The non-synonymous mutations in *HXT2* and *CIT1* may function in the presence of mutated *MTH1* alleles and could be related to an altered central carbon metabolism in order to ensure production of cytosolic acetyl-CoA in the Pdc negative strain.

**Electronic supplementary material:**

The online version of this article (doi:10.1186/s12934-015-0305-6) contains supplementary material, which is available to authorized users.

## Background

*Saccharomyces cerevisiae* is an important cell factory widely used for the production of beer, bread, wine, bioethanol, nutraceuticals, chemicals and pharmaceuticals [1–5]. When grown on glucose, the majority of the glycolytic flux is directed towards ethanol due to the so-called Crabtree effect in *S. cerevisiae*. The only strategy to eliminate ethanol production that has worked so far is removing pyruvate decarboxylase activity so that pyruvate cannot be converted to acetaldehyde, the direct precursor of ethanol [6]. In *S. cerevisiae*, pyruvate decarboxylase is encoded by three structural genes, *PDC1*, *PDC5* and *PDC6* [7–9]. However, pdc triple deletion mutants (*pdc1∆ pdc5∆ pdc6∆*, also called Pdc negative strains) cannot grow on glucose as the sole carbon source [10]. The metabolic responses of Pdc negative strains, growth requirements, and growth recovery by threonine aldolase (encoded by *GLY1*) over-expression all suggested that the growth defect of Pdc negative strain on glucose was due to the lack of cytosolic acetyl-CoA for biosynthesis of cellular biomolecules, especially lipids.

Interestingly, the Pdc negative strains are still sensitive to high glucose concentrations even when supplemented with a C_2_ source or with *GLY1* over-expression [6, 11]. van Maris et al. performed directed evolution of a Pdc negative strain on glucose, yielding the ‘C_2_-independent, glucose-tolerant, and pyruvate-hyperproducing’ strain TAM [12]. In the TAM strain, a *MTH1* allele with a 225 bp internal deletion (*MTH1*-*∆T*) was identified, and was attributed with restoring growth of the Pdc negative strain on glucose [13]. However, when introducing the *MTH1*-*∆T* allele into an un-evolved Pdc negative strain, the growth rate (μ_max_ = 0.10 h^−1^) was slower in minimal medium with 2% glucose, compared to the TAM strain (μ_max_ = 0.20 h^−1^), indicating the possible presence of additional advantageous genetic changes in the TAM strain besides *MTH1*-*∆T*.

Mth1 functions as a negative transcriptional regulator in the glucose signaling pathway together with other regulators, i.e. Snf3, Rgt2, Std1, Rgt1. Several other *MTH1* alleles have been identified in selections of glucose or catabolite repression suppressors using glucose sensitive mutants [14–18]. The *MTH1* alleles seemed to be able to resolve the sensitivity to glucose in these mutants. Previous studies have shown that these *MTH1* alleles reduced glucose transport by repressing the transcription of several hexose transporter genes (*HXT*s) [12, 14, 16, 17], and that over-expression of *MTH1* had similar effects [13]. It has been proposed that *MTH1*-*∆T* resulted in a decreased degradation of Mth1 [13], which could be related to putative PEST sequences (usually present in proteins with short intracellular half-life) and a target site for phosphorylation by casein kinase Yck1 [19] situated inside the deleted region. Mth1 or its paralog Std1 interacts with Rgt1, which also interacts with other transcription factors and binds the promoters of hexose transporter genes [20, 21]. The decreased degradation of Mth1 could prevent the phosphorylation of Rgt1, required for its release from the promoters of several hexose transporters [21]. The decreased degradation of Mth1 resulted from the *MTH1*-*∆T* allele could therefore repress the transcription of hexose transporter genes even during growth on glucose.

In this study, a Pdc negative strain PDC-E1 (*MATaura3*-*52 his3*-*Δ1 pdc1Δ pdc5Δ pdc6Δ*) was evolved in glucose medium via serial transfer in 3 independent culture lines. Genomic DNA sequencing of these three evolved Pdc strains identified mutations in *MTH1* as well as in *HXT2* [22], *CIT1* [23] and *RPD3* [24]. The fact that the mutated *MTH1* alleles were also identified in our evolved Pdc negative strains, indicated again that *MTH1* might be an important target for relieving high glucose repression. In order to understand the roles of these genetic changes in the evolved strains, the effects of the mutations in mutated genes, *MTH1*, *HXT2*, *CIT1* and *RPD3*, were investigated using homology analysis of their protein sequences and published crystal structure models of homologous proteins, and possible mechanisms were proposed and discussed. Although the speculations regarding the possible mechanisms in evolved Pdc negative strains still require further investigations, they may be useful and helpful for metabolic engineering strategies on Pdc negative strains.

## Results and discussion

### Construction of Pdc negative strains and strain evolution

CEN.PK 113-5D (*MATaura3*-*52*) and CEN.PK 110-10C (*MATαhis3*-*Δ1*) were used as background strains for the construction of Pdc negative strains (Additional file 1: Figure S1). A collection of triple deletion mutants were obtained using a bipartite deletion strategy (Additional file 1: Figure S2), strain crossings, and tetrad segregations, including PDC-E1 (*MATaura3*-*52 his3*-*Δ1 pdc1Δ pdc5Δ pdc6Δ*) (Table 1).

**Table 1:** Strains used in this study

Strain name	Genotype or description
CEN.PK 113-5D	*MATa* *ura3*-*52*
CEN.PK 110-10C	*MATα* *his3*-*Δ1*
PDC-A1	*MATa* *ura3*-*52 pdc5Δ*
PDC-A2	*MATa* *ura3*-*52 pdc1Δ*
PDC-A3	*MATa* *ura3*-*52 pdc6Δ*
PDC-B1	*MATa* *ura3*-*52 pdc1Δ pdc5Δ*
PDC-B2	*MATα ura3*-*52 pdc1Δ pdc6Δ*
PDC-C1	*MATa* *ura3*-*52 pdc1Δ pdc5Δ pdc6Δ*
PDC-C2	*MATα* *ura3*-*52 pdc1Δ pdc5Δ pdc6Δ*
PDC-D1	*MATa* *his3*-*Δ1 pdc1Δ pdc5Δ pdc6Δ*
PDC-D2	*MATα* *his3*-*Δ1pdc1Δ pdc5Δ pdc6Δ*
PDC-E1	*MATa* *ura3*-*52 his3*-*Δ1 pdc1Δ pdc5Δ pdc6Δ*
PDC-E2	*MATα* *ura3*-*52 his3*-*Δ1 pdc1Δ pdc5Δ pdc6Δ*
PDC-E1A	PDC-E1 adaptively evolved in glucose
PDC-E1B	PDC-E1 adaptively evolved in glucose
PDC-E1C	PDC-E1 adaptively evolved in glucose
M81-11	PDC-E1, *mth1::MTH1* ^*81D*^
M81C	M81-11, *cit1*::*amdSYM*

PDC-E1 (*MATaura3*-*52 his3*-*Δ1 pdc1Δ pdc5Δ pdc6Δ*), E1 for short, was evolved in 3 independent culture lines in YPD medium with gradually reduced ethanol concentration. Once fast-growing, glucose tolerant strains were obtained in YPD media, they were further evolved for C_2_ carbon source-independent and faster growth in minimal medium with 2% glucose.

**Fig. 1:**
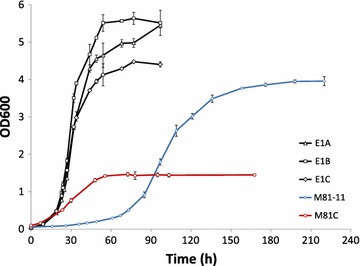
Growth profiles of the evolved Pdc negative strains and reverse engineered strains M81-11 and M81C. E1A, E1B and E1C are three evolved Pdc negative strains. M81-11 is a Pdc negative strain with a point mutation in *MTH1* (*ura3*-*52 his3*-*Δ1 pdc1∆ pdc5∆ pdc6∆ mth1::MTH1* ^*81D*^). M81C is a M81-11 mutant with the *CIT1* deletion. The cultivations were performed in minimal medium with 2% glucose in replicate. *Error bars* represent ±standard errors.

At the end of the adaptive evolution, three independently evolved E1 strains were obtained. We refer to these as PDC-E1A, PDC-E1B, and PDC-E1C (E1A, E1B, E1C for short, respectively). E1A, E1B, E1C could grow in minimal medium with glucose as the sole carbon source, with maximum specific growth rates of 0.138, 0.148, 0.141 h^−1^, respectively (Fig. 1).

### Genomic sequencing results of the unevolved and evolved Pdc negative strains

Genome sequencing of the parental strain E1 and its evolved strains (E1A, E1B, E1C) was performed to study the genetic changes that occurred during the adaptive evolution. The raw sequencing data were filtered and trimmed to remove adaptor sequences and sequence ends with a quality score below 20. The filtered reads were mapped to the CEN.PK 113-7D reference genome. In the E1A strain, three single nucleotide variants (SNVs) in coding regions representing non-synonymous mutations were identified, as listed in Additional file 2: Table S2. In the E1B strain, 11 SNVs in coding regions representing non-synonymous mutations were identified. In the E1C strain, six SNVs in coding regions representing non-synonymous mutations, one chromosomal regional deletion, one mitochondrial regional deletion and one single nucleotide insertion were identified.

Among all genes with SNVs, three genes, *MTH1*, *CIT1*, *HXT2,* were found to carry point mutations in all three evolved strains. And one gene, *RPD3*, was found to carry point mutations in two of the evolved strains. The mutations of the proteins encoded by the four genes are listed in Table 2. It is interesting to note that the same mutations occurred in more than one strain for two loci, A81D in Mth1 and W466* in Hxt2, and that two mutations affected adjacent amino acids, P176Q and H175R in Cit1.

**Table 2:** Point mutations in evolved E1 strains

Name	Description	Evolved strains		
		E1A	E1B	E1C
Mth1	Negative regulator of the glucose-sensing signal transduction pathway	A81/D	I85/S	A81/D
Cit1	Mitochondrial citrate synthase	P176/Q	M84/V	H175/R
Hxt2	High-affinity glucose transporter of the major facilitator superfamily	W466/*	G75/R	W466/*
Rpd3	Histone deacetylase, component of both the Rpd3S and Rpd3L complexes		85F/I	196A/V

### Integration of *MTH1*^*81D*^ in non-evolved Pdc negative strain E1

As previously reported by Oud et al. [13], a 225 bp in-frame internal deletion (corresponding to amino acids 57–131) in *MTH1* was identified in their evolved Pdc negative strain TAM, which was demonstrated to be responsible for relieving glucose sensitivity in the Pdc negative mutant. Among the previously identified *MTH1* alleles affecting glucose sensing, e.g. *BPC1-1*, *DGT1*-*1*, *HTR1*-*5*, *HTR1*-*19* and *HTR1*-*23*, two mutations in codon 85 (I85N, I85S) and one mutation in codon 102 (S102G) were found, and it was also found that the S102G mutation could reinforce the mutations in codon 85 although having no effects by itself [16, 17]. Since the *MTH1*^*85S*^ allele has already been confirmed to suppress glucose repression, we chose to validate the *MTH1*^*81D*^ allele through reverse engineering in this study, to investigate if it has similar effects of suppressing glucose repression like other alleles.

The *MTH1*^*81D*^ allele was integrated into the *MTH1* locus of the non-evolved E1 strain, resulting in strain M81-11. The growth profile in minimal medium with 2% glucose is shown in Fig. 1 with the maximum specific growth rate of 0.053 h^−1^. The fact that *MTH1*^*81D*^ by itself could restore the growth of the Pdc negative strain on glucose suggested it had the similar effects on glucose repression alleviation. However, the maximum specific growth rate was not as high as those of the evolved strains E1A, E1B, E1C. The *MTH1*^*81D*^ mutation contributes around 35% of the maximum specific growth rate in the evolved E1 strains, indicating there are likely other genetic changes that contribute to their growth recovery.

### Analysis of the mutations in Mth1 and their possible effects

Even though the N-terminal part of Mth1 appears to be important in glucose repression it is not yet clear why. To gain sequence-function insight into Mth1 protein, a bioinformatics approach was undertaken.

**Fig. 2:**
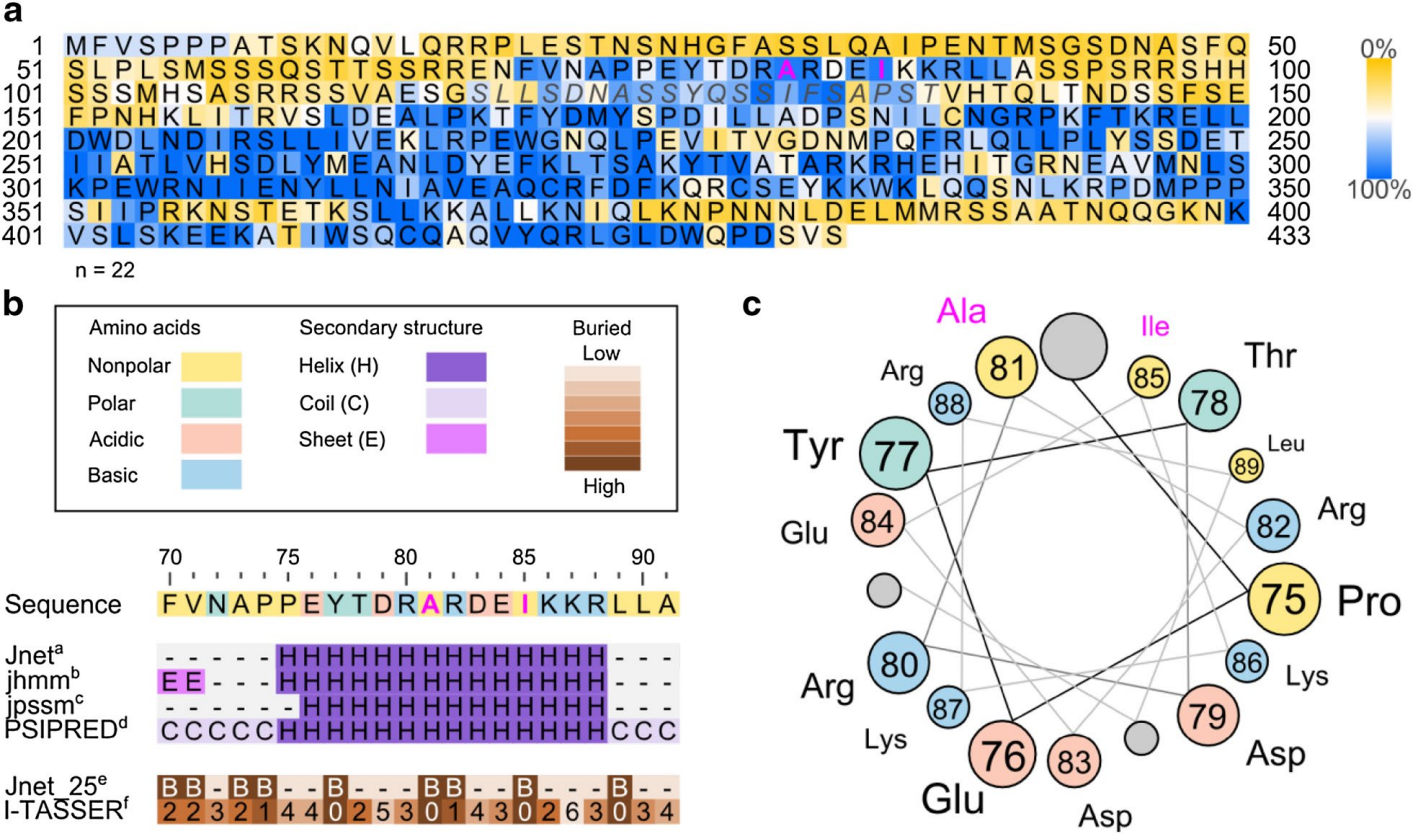
Mapping and analysis of Mth1 mutations. *Magenta text* indicates positions for non-synonymous mutations identified in this study. **a** Result of a multiple alignment using homologous sequences (n = 22). The Mth1 sequence of *S. cerevisiae* is shown with *colored* conservation levels. *Yellow* indicates low conservation, *white* intermediate, and *blue* high. *Gray* and *italic text* indicates the phosphorylation region of casein kinase Yck1. **b** Analysis of the 22 amino acids from the conserved region at position 70–91 and secondary structure predictions using six different prediction programs [25, 26]. **c** A helix wheel representation of amino acids predicted to form an alpha-helix (codon 75–89). The amino acid types are *colored* as in **b**.

Alignment results of Mth1 from *S. cerevisiae* and 22 other homologous sequences of Mth1 and Std1 from unicellular fungi revealed that A81 and I85 are positioned on an ‘island’ composed of 22 highly conserved amino acids from codon 71–91 (Fig. 2a). Since no protein crystal structures of Mth1 or homologs thereof are available, a secondary structure prediction was performed for the conserved ‘island’ using four prediction tools [25] (Fig. 2b), which indicated an alpha amphipathic helix in this region with the hydrophilic side facing solvent and the hydrophobic side facing the core of the protein (Fig. 2c). The putative helix is likely initiated by the structurally rigid prolines at codon 74 and 75, and Y77, A81, I85 and L89 are buried away from the solvent based on two prediction tools [26] (Fig. 2b). Therefore, it is possible that A81 and I85, together with Y77 and L89, play a structural role in anchoring a highly conserved alpha helix to the Mth1 surface through hydrophobic interactions. The A81D, I85S or I85N mutations, representing non-polar to polar amino acid changes, would disrupt these interactions and may therefore cause structural changes in the protein.

It is interesting to note that another conserved ‘island’ from codon 118–137 is the identified target region for phosphorylation by Yck1 [19]. Therefore, it is reasonable to speculate that the predicted helix structure within the conserved island from codon 71–91 might play an important part in the function of Mth1.

### Transcriptional analysis of hexose transporter genes

In order to understand the effects of the *MTH1*^*81D*^ allele on transcription of genes encoding hexose transporters, transcription analysis were performed on *HXT1*-*7* using qPCR in reverse engineered strain M81-11 and wild type strain CEN.PK 113-11C [27].

**Fig. 3:**
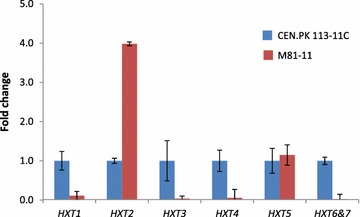
Transcription analysis of *HXT*s (*HXT*1-7) in the M81-11 and wild type strain CEN.PK 113-11C. Cells for transcription analysis were harvested at exponential phase (OD_600_ ~ 1). The expression levels of *HXT*s in wild type strain were set as 1.

Compared with wild type strain, the expression levels of *HXT1*, *HXT3*, *HXT4* and *HXT6&7* were much lower in M81-11, i.e. around 9 fold, 25 fold, 15 fold, and 40 fold lower, respectively (Fig. 3). The expression level of *HXT5* did not differ much between the wild type strain and the M81-11. However, the expression level of *HXT2* was around 3 fold higher in the M81-11 strain, which was quite a different pattern from the other *HXT*s. The different expression patterns of *HXT*s in M81-11 is consistent with those in the TAM strain [12], suggesting that they are regulated by Mth1 differently, which is consistent with earlier findings [28].

According to the bioinformatics analysis and its homolog protein structure, the mutations identified in Hxt2 in evolved Pdc strains were predicted to impair or completely abolish its activity to transport glucose due to the structural changes around the transport channel (Additional file 3: Figure S5). Therefore, it is possible that the glucose transport by Hxt2 was decreased due to the identified mutations, even with an increase in its transcription level, which resulted from the mutations in Mth1.

### Deletion of *CIT1* in the M81-11 strain

**Fig. 4:**
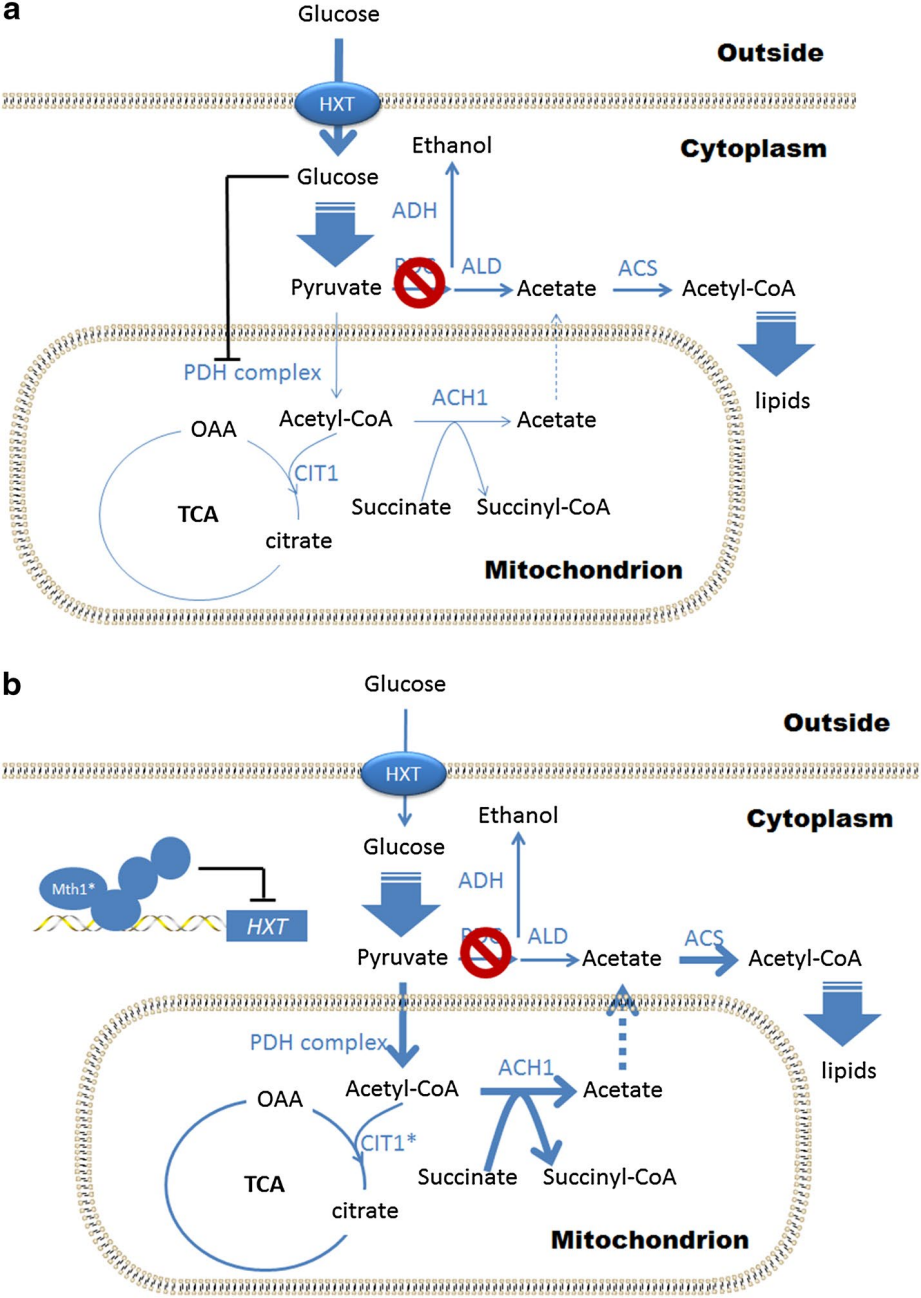
A simple illustration for possible roles of mutated proteins in the evolved Pdc negative strains. The *blue solid arrows* represent the reactions catalyzed by the enzymes, which are indicted in *blue text*. The *blue dash line* represents the transportation between different subcellular organelles. The *black lines* with a *bar* at one end represent the repression or inhibition. The *red circles* represent the block due to Pdc deletions. **a** Simplified acetyl-CoA metabolism in the parental Pdc negative strain. The PDH complex and TCA cycle enzymes is repressed by high glucose uptake via hexose transporters (HXT). **b** Simplified acetyl-CoA metabolism in evolved Pdc negative strain with point mutated Mth1 (Mth1*) and Cit1 (Cit1*). Glucose uptake via HXT decreases in the presence of Mth1*, resulting in derepression of the PDH complex and TCA cycle enzymes. Cit1* with predicted decreased activity allows more mitochondrial acetyl-CoA convert to acetate by Ach1, which can be transported to the cytosol and converted to acetyl-CoA there.

A recent study revealed that cytosolic C_2_ can be derived from mitochondrial C_2_ in the form of acetate involving mitochondrial CoA transferase Ach1 in Pdc negative strains [29]. The Cit1 mutations might be connected with cytosolic C_2_ supply, since Cit1 competes with Ach1 for mitochondrial acetyl-CoA (Fig. 4). The mutated Cit1 has potentially decreased activity, predicted by the analysis concerning possible structural disruptions of the identified mutations (Additional file 3: Figure S6), and this might allow more acetyl-CoA being converted to acetate by Ach1, thus providing more acetate for acetyl-CoA biosynthesis in the cytosol.

To test the hypothesis, *CIT1* was deleted in the M81-11 strain, resulting in the strain M81C. Compared to M81-11, the strain M81C showed a higher maximum specific growth rate of 0.069 h^−1^ when cultured in minimal glucose medium (Fig. 1), indicating the *CIT1* deletion did have a positive effect on cell growth. When *CIT1* was deleted, the incomplete TCA cycle resulted in inefficient pyruvate dissimilation, and the accumulated pyruvate (data not shown) caused the low pH (<3) in the cell culture. Therefore, a lower final OD_600_ was observed in M81C compared to M81 (Fig. 1).

### Possible evolving mechanisms in Pdc negative strains

According to the bioinformatics analysis of the point mutations on their corresponding proteins (Additional file 3), all the point mutations occurred at the highly conserved residues and might cause structural disruptions and result in enzyme activity decreases or complete activity abolishment. Therefore, possible mechanisms were proposed for how the evolved Pdc negative strains have acquired a faster growth phenotype.

The growth defect of a Pdc negative strain on glucose [10] was previously attributed to the lack of cytosolic acetyl units, since acetyl-CoA cannot be transported between different subcellular compartments freely and ethanol or acetate supplementation or *GLY1* over-expression could restore its growth on glucose [6, 11, 30]. However, the growth recovery of the Pdc negative strain with *MTH1*-*∆T*, which does not in itself lead to cytosolic acetyl-CoA provision, indicated the presence of native pathways to cytosolic C_2_ compounds. This was recently identified in the form of acetate, converted from mitochondrial acetyl-CoA by CoA transferase (or acetyl-CoA hydrolase, encoded by *ACH1*) [29]. Although Ach1 is involved in channeling acetyl-CoA from the mitochondria to the cytosol, it is only functional under glucose derepressed conditions and this route can also be blocked by limited acetyl-CoA availability due to the stringent regulation of the PDH complex in *S. cerevisiae* (Fig. 4a), e.g. via post-transcriptional phosphorylation of the Pda1 subunits [31] or transcriptional repression of the Lpd1 subunits [32]. In the evolved Pdc negative strains, the mutations in Mth1 seemed to play the most critical role, since the single mutation *MTH1*^*81D*^ alone was here shown to improve the growth of a Pdc negative strain on glucose as well as the earlier reported truncation *MTH1*-*∆T*. According to homology analysis of Mth1 and predictions of the conserved ‘island’ (codon 71–91), where the mutations are located, A81 and I85 may play a critical role in maintaining the alpha helix structure formed within it, and thus probably affect the function of Mth1. However, a crystal structure of Mth1 would be needed to validate these predictions.

The *MTH1* alleles seem to decrease glucose uptake transport by alleviated repression of transcribing several *HXT*s, especially *HXT1* and *HXT3* [13–18], which was also found in the TAM strain (evolved Pdc negative strain, with *MTH1*-*∆T* allele) by van Maris et al. [12]. Moreover, a deletion within *HXT3* (~1,000 bp) was also found in the evolved strain E1C, which would undoubtedly destroy its activity to transport glucose. A previous study suggested that the rate of glucose transport determines the strength of glucose repression [33], and the attenuated glucose uptake therefore likely resulted in a generally reduced glucose repression in the evolved strains. Thus, the transcription of many genes, which are normally repressed by glucose, could probably be partially de-repressed despite the high extracellular glucose concentration, e.g. genes encoding mitochondrial enzymes. Therefore, in the evolved strains with mutated *MTH1*, it is possible that the C_2_ supply from the mitochondria to the cytosol via Ach1 route was no longer blocked (Fig. 4b).

Hxt2, a high-affinity glucose transporter, is usually found to function under low glucose concentrations and its transcription is repressed by high glucose and induced by low glucose [34, 35]. The mutations in Hxt2 seemed to make no sense in high glucose medium (2% glucose) used in this study. One possible hypothesis would be that the effects of the mutated Mth1 might be quite complicated. Transcriptional analysis of the TAM strain showed decreased transcription of *HXT1*, *HXT3*, *HXT4*, *HXT6* and *HXT7*, and increased transcription of *HXT2* and *HXT5*, although this might resulted from other genetic changes besides *MTH1*-*∆T* [12]. Previous studies with other *MTH1* alleles revealed significant decreased transcription of *HXT1* and *HXT3*, a large increase in *HXT7* and nearly no change in *HXT2* [16, 17]. All these results suggested that *MTH1* alleles might have different effects in regulating expression of hexose transporters with low affinity compared to those with high affinity. According to previous results, the mutated *MTH1* might result in an unchanged or increased transcription level of *HXT2*, which has been confirmed by our qPCR results (Fig. 3). The mutations in Hxt2 might cause structural disruptions based on our predictions (Additional file 3: Figure S5), and may therefore further reduce the glucose transport.

As mentioned above, the role of the mutated Cit1 might also be connected with cytosolic C_2_ supply via the mitochondria. Since C_2_ carbon supply in the cytosol seemed to be a limiting step for the growth of Pdc negative strains [29]. The mutated Cit1 with decreased activity might further improve the strain growth on glucose in the presence of the mutated Mth1, since Cit1 competes with Ach1 for mitochondrial acetyl-CoA. The complete disruption of *CIT1* in the M81-11 strain increased the maximum specific growth rate, which supports this speculation.

Rpd3 usually functions in the form of a complex together with other proteins to regulate gene transcription, silencing and many other processes by histone deacetylation [36–38]. More and more studies suggest that histone acetylation and deacetylation regulate gene transcription in complex and comprehensive ways [39]. Although the mutations in Rpd3 might result in its decreased activity due to the possible structural disruptions (Additional file 3: Figure S7), it is still difficult to speculate about their role in the evolved strains. A previous study found that histone acetylation and deacetylation was directly regulated by nucleocytosolic acetyl-CoA abundance [40]. One possible speculation would be that the Rpd3 mutations might be related to cytosolic acetyl-CoA abundance, but this will require further investigation.

## Conclusions

In this study, a Pdc negative strain was adaptively evolved in glucose media via serial transfer, and evolved Pdc negative strains were shown to grow on glucose as the sole carbon source. Genomic DNA sequencing results of the parental Pdc negative strain and its corresponding evolved strains revealed four genes which carried point mutations in at least two of the evolved strains. The mutations in these four genes seemed to be related to the cytosolic acetyl-CoA supply. These findings will be useful for the fundamental understanding of acetyl-CoA metabolism in *S. cerevisiae*, as well as strain development for biochemical production as cell factories.

## Methods

### Strain construction

*PDC1*, *PDC5* and *PDC6* were deleted using a bipartite strategy [41] (Additional file 1: Figure S1). Sequences upstream and downstream of the individual genes were amplified using primers 1–12 listed in Additional file 1: Table S1. Two overlapping fragments of the *kanMX* resistance marker cassette flanked by *loxP* sites were PCR amplified from plasmid pUG6 [42] using primers 13–16 listed in Additional file 1: Table S1. The two fused PCR fragments for each gene deletion were transformed into yeast using the lithium acetate method [43]. After each gene deletion, the *kanMX* marker cassette was looped out via Cre recombinase mediated recombination between the two flanking *loxP* sites using plasmid pUC47 or pUG62 as described previously [42]. Each gene deletion was confirmed using primers 17–22 listed in Additional file 1: Table S1.

*PDC1*, *PDC5*, and *PDC6* were consecutively deleted in two different background strains, CEN.PK 113-5D (*MATaura3*-*52*) and CEN.PK 110-10C (*MAT*α *his3*-*Δ1*) [27]. Together with strain crossing and tetrad segregation, a collection of triple deletion mutants was constructed carrying different auxotrophic markers: *ura3*-*52*, *his3*-*Δ1* or *ura3*-*52 his3*-*Δ1*. The mating type of these pdc negative mutants was determined using primers 35–37 listed in Additional file 1: Table S1.

To create a Pdc-negative strain with a point mutation in *MTH1*, an *MTH1*^*81D*^ construct was created and used to replace the normal *MTH1* gene in strain PDC-E1 (*MATaura3*-*52 his3*-*Δ1 pdc1Δ pdc5Δ pdc6Δ*), as shown in Additional file 1: Figure S3, resulting in the M81-11. *MTH1*^*81D*^ with upstream and downstream sequences was amplified using primers 23–26, and the sequence downstream of *MTH1*^*81D*^ was amplified using primers 27–28. Two overlapping fragments of the *amdSYM* marker cassette were PCR amplified from plasmid pUG-amdSYM (obtained from Euroscarf, Accession Number: P30669) using primers 29–32. The two fused PCR fragments for integration were transformed into PDC-E1 using the electroporation method as described in [44]. The integration of *MTH1*^*81D*^ and *amdSYM* was confirmed using primers 33–34. The *amdSYM* marker was looped out via selection on counter-selective plates. The replacement of *MTH1*^*81D*^ was confirmed by sequencing PCR product amplified by primers 33–34.

*CIT1* was deleted in strain M81-11 using the same strategy as described in *PDC1* deletion (Additional file 1: Figure S2), in which the *amdSYM* marker was used instead of the *kanMX* marker, resulting the strain M81C. Sequences upstream and downstream of the individual genes were amplified using primers 38–41 listed in Additional file 1: Table S1. Two overlapping fragments of the *amdSYM* marker cassette were PCR amplified from plasmid pUG-amdSYM using primers 29–32 listed in Additional file 1: Table S1. The two fused PCR fragments for integration were transformed into M81-11 using the electroporation method as described in [44]. The gene deletion was confirmed using primers 42–43 listed in Additional file 1: Table S1.

### Medium and culture conditions

Cultivations were performed at 30°C in YP medium with 2% glucose (YPD) or 2% ethanol (YPE). Selections for transformants containing the *kanMX* marker were performed on YPD or YPE plates supplemented with 200 mg/L G418 sulfate (Formedium Ltd., Hunstanton, UK). Selections for transformants carrying the *amdSYM* marker were performed on SM-Ac plates with 2% ethanol instead of glucose, and the counter selections were performed on SM-Fac plates with ethanol as carbon source, as described in [45]. A diploid resulting from two haploids of different mating type was obtained on agar plates containing a synthetic medium consisting of yeast nitrogen base (Formedium Ltd.), complete supplement mixture w/o uracil or histidine (Formedium Ltd.), and 2% (v/v) ethanol (SE-ura-his). Tetrad dissections were performed on YPE agar plates.

Cultivations for strain characterization were performed at 30°C in triplicate in 100 mL shake flasks with 40 mL defined minimal medium. The defined minimal medium for cultivation was composed of 20 g/L glucose, 7.5 g/L (NH_4_)_2_SO_4_, 14.4 g/L KH_2_PO_4_, 0.5 g/L MgSO_4_∙7H_2_O, 2 mL/L trace metal solution, 1 mL/L vitamin solution, with the pH adjusted to 6.5 by adding 2 M NaOH. The final concentrations of trace metal elements and vitamins were previously described in [46]. The minimal medium was supplemented with 40 mg/L uracil or 40 mg/L histidine when required.

### Determination of biomass and extracellular metabolites

Biomass was determined by optical density (OD_600_) measurement at a wavelength of 600 nm with a GENESYS™ 20 Visible spectrophotometer (Thermo Electron Scientific, Madison, USA). Glucose, ethanol, glycerol, pyruvate and formate concentrations were determined in culture supernatants by high-performance liquid chromatography (Dionex-HPLC, Sunnyvale, CA, USA) equipped with UV detector and RI detector using a Bio-Rad HPX 87H column (Bio-Rad, Hercules, CA, USA). The HPLC was operated at 45°C with 5 mM H_2_SO_4_ as mobile phase at a flow rate of 0.6 mL min^−1^.

### Adaptive evolution of Pdc negative strain in glucose medium via serial transfer

The adaptive evolution of PDC-E1 (*MATaura3*-*52 his3*-*Δ1 pdc1Δ pdc5Δ pdc6Δ*) towards growth on glucose as the sole carbon source were performed in three independent culture lines in 100 mL shake flasks with 20 mL medium at 30°C, which involved three phases (Additional file 1: Figure S4). In the first phase, strains were cultivated in YP medium containing 1.4% glucose and 0.6% ethanol and then serially transferred every 48 or 24 h using YP medium with gradually decreased ethanol concentration for 8 days. Subsequently, the strains were evolved in YPD medium for 15 days and transferred with passage every 48 or 24 h. Finally the strains were transferred into minimal medium containing 2% glucose as the sole carbon source and evolved for increased growth by serial transfer every 48 or 24 h for 39 days. And single clone isolates were obtained from the last shake flasks, and designated as PDC-E1A, PDC-E1B, and PDC-E1C.

### Genomic DNA extraction and sequencing procedures

PDC-E1 and their evolved strains were cultured in 10 mL YPE medium at 30°C, and cells were harvested during exponential phase for genomic DNA extraction. Genomic DNA was extracted using the Genomic DNA buffer set (QIAGEN, Hilden, Germany) and QIAGEN Genomic-tip 500/G. The DNA samples were prepared for sequencing using the Illumina DNA TruSeq protocol, with an insert size of 650 bp. The sequencing was performed multiplexed on an Illumina MiSeq using the version 2 chemistry (2 × 250 bp, paired reads).

### Analysis of genome sequencing results

The raw sequencing data was filtered and trimmed to remove adaptor sequences and sequence ends with a quality score below 20. The filtered reads were mapped to the CEN.PK 113-7D reference genome (http://cenpk.tudelft.nl) using the mapper MosiakAligner version 2.1.32 (http://code.google.com/p/mosaik-aligner/) with a hash size of 15. Sites with potential indels were detected and realigned using the tools from the Genome Analysis Toolkit (GATK, version 2.3.9) [47], RealignerTargetCreator and IndelRealigner and potential PCR duplicates were eliminated using the MarkDuplicates tool from Picard version 1.100. (http://picard.sourceforge.net). The number of mappable reads after post-processing ranged from 2.9 to 5.2 million resulting in a mapped coverage spanning from approximately 50× to over 100× in the different samples. Variant calling (single nucleotide variants and small indels) was performed using GATK UnifiedGenotyper, and annotation of the detected variants was performed using SnpEff version 3.4 (http://snpeff.sourceforge.net). Finally, the alignments were visually inspected for all detected variants, in order to eliminate obvious false positives caused by incorrect alignments.

### qRT-PCR procedures and gene expression analysis

Cells were cultured in shake flasks using minimal medium with 2% glucose in triplicate, and harvested for gene expression analysis at exponential growth phase (OD_600_ ~ 1.0) by centrifugation at −20°C, quenched by liquid nitrogen, and stored at −80°C for use. Total RNA was isolated using RNeasy Mini Kit (QIAGEN), which was then processed to obtain fragmented cDNA using a QuantiTect Reverse Transcription Kit (QIAGEN). 2 μL of the synthesized cDNA (corresponding to 100 ng RNA) was used as the template for the qPCR reaction to a final reaction volume of 20 μL, using a DyNAmo Flash SYBR Green qPCR Kit (Thermo Scientific, USA). Quantitative RT-PCR was performed on Stratagene Mx3005P (Agilent Technologies, USA). The thermocycling program consisted of one hold at 95°C for 15 min, followed by 40 cycles of 10 s at 95°C and 20 s at 60°C, and a final cycle of 1 min at 95°C, 30 s at 55°C and 30 s at 95°C.

Primers for real-time PCR (Additional file 1: Table S1) were designed using Primer3 (http://bioinfo.ut.ee/primer3-0.4.0/) with melting temperature (T_m_) around 60°C. *ACT1*, a housekeeping gene, was selected as the reference gene [48]. Final concentration of primers used was 0.5 mM in 20 μL qPCR reactions. Due to nearly identical sequences of *HXT*6 and *HXT7*, they used the same pair of primers for real-time PCR. Therefore, the transcription analysis result for *HXT*6 and *HXT7* was their sum-up.

### Bioinformatics analysis

The protein sequence of each of the four *S. cereviciae* proteins Cit1 (UniProt# P00890), Hxt2 (UniProt# P23585), Rpd3 (UniProt# P32561) and Mth1 (UniProt# P35198) was separately used to query the NCBI database with the basic local alignment search tool (BLAST). To eliminate sequences with low identity to the *S. cerevisiae* sequences, the BLAST results were filtered to retain only those with more than 40% identity to the *S. cerevisiae* enzymes. The 40% cutoff was arbitrary set and is intended to limit the analysis to sequences which can reasonably be said to have the same function as the *S. cerevisiae* enzyme. Sequences were also filtered such that anything more than 95% identical to anything else in the dataset was removed. This was done to reduce bias, should there be a large number of deposited sequences of the same gene from the same organism. The remaining sequences were aligned using MUSCLE [49]. The resulting alignment was used to compute the conservation of each amino acid in the *S. cerevisiae* proteins compared to the filtered BLAST results. The computed conservation values were used to color-code sequence representations of the *S. cerevisiae* proteins. Homology models were generated for three of the four proteins using the Swiss-Model repository [50]. For Mth1 no homology model could be made as there is no crystal structure of a homologous protein. An alternative approach was therefore used where the secondary structure of the mutated region was predicted. This prediction was used in the data analysis.

## Additional files

**Additional file 1.** Primers and strategies for Pdc negative strain constructions and strain evolution.
**Additional file 2.**  Genome sequencing results of Pdc negative strain E1 and its evolved strains E1A, E1B, E1C.
**Additional file 3.** Bioinformatics analysis of the mutations in Hxt2, Cit1 and Rpd3.
