# Supplementary material for: Adaptive mutations in sugar metabolism restore growth on glucose in a pyruvate decarboxylase negative yeast strain
Source: Microb Cell Fact. 2015 Aug 8;14:116. doi: 10.1186/s12934-015-0305-6 (PMC4529725; doi:10.1186/s12934-015-0305-6)
Supplement: Supplementary file 1 — Additional file 1. Primers and strategies for Pdc negative strain constructions and strain evolution. [file 12934_2015_305_MOESM1_ESM.docx]

Table S1. Primers used in this study and their sequences

| Primer # | Primer name | Sequence 5´-3´ |
| --- | --- | --- |
| 1 | pdc1-up-fw | TACTTGCTATCGTTCAACAC |
| 2 | pdc1-up-rev | CAGCGTACGAAGCTTCAGTGCGTGAGGTTATGAGTAG |
| 3 | pdc1-dw-fw | GTGATATCAGATCCACTAGTACCAACGCTAAGCAATAAG |
| 4 | pdc1-dw-rev | CCTTGGTTCCACTAATTCATC |
| 5 | pdc5-up-fw | TTAGGCATAATCACCGAAGA |
| 6 | pdc5-up-rev | CAGCGTACGAAGCTTCAGGAGAGGAAAGGACTTACTACA |
| 7 | pdc5-dw-fw | GTGATATCAGATCCACTAGTTCTGTCCTGTCTTCCAG |
| 8 | pdc5-dw-rev | GGTGCTCTACTGGTGATT |
| 9 | pdc6-up-fw | ACATCTTCCAAGCATCTCAT |
| 10 | pdc6-up-rev | CAGCGTACGAAGCTTCAGGAATCGCACCATATCCCTTA |
| 11 | pdc6-dw-fw | GTGATATCAGATCCACTAGCGTTATCGCCGTGAATTAC |
| 12 | pdc6-dw-rev | TTGGTTGTAGATGGTGGTG |
| 13 | kanMX_1_fw | CTGAAGCTTCGTACGCTG |
| 14 | kanMX_1_rev | TCACCATGAGTGACGACTGA |
| 15 | kanMX_2_fw | TTCCAACATGGATGCTGAT |
| 16 | kanMX_2_rev | CTAGTGGATCTGATATCAC |
| 17 | pdc1-up-check | GTGATGAGGCTCGTGGAA |
| 18 | pdc1-dw-check | CGAGGTGTCTAGTCTTCTATT |
| 19 | pdc5-up-check | ACTGCCATCACTAGAGAAGA |
| 20 | pdc5-dw-check | TTGTTCGGAGTTCCATTCAT |
| 21 | pdc6-up-ck | TCGGTTCCTCATCATCTCT |
| 22 | pdc6-dw-ck | TCTTGTCCTTACGGTCTCT |
| 23 | mth_up_fw | GCTGCCTCAATCTCCATTA |
| 24 | Mth1_81_rev | CTCATCTCTAtCTCTATCAGTGTACTCCGGA |
| 25 | Mth1_81_fw | CTGATAGAGaTAGAGATGAGATTAAAAAAAG |
| 26 | mth1_dw_rev_n1 | GTATTCTGGGCCTCCATGTCGCTGTGCTCAACTACCAA |
| 27 | mth1_in_fw2_n1 | GAATGCTGGTCGCTATACTGATGCCTCGTCTTATCAATCA |
| 28 | mth_dw_rev | GCTGTGCTCAACTACCAA |
| 29 | amdS-F1 | GACATGGAGGCCCAGAATAC |
| 30 | amdS-R1 | CAGTATAGCGACCAGCATTC |
| 31 | amdS_in_F | TCTTGTGGTGGTTCTTCTG |
| 32 | amdS_in_R | GAGTGATTGGAGCGATGAT |
| 33 | mth1_up_fw1 | CCGAGACTTACTTGGACTT |
| 34 | mth1_dw_rev1 | CATTGTTGTATTGTGCTGTG |
| 35 | MAT | AGTCACATCAAGATCGTTTATGG |
| 36 | MAT a | ACTCCACTTCAAGTAAGAGTTTG |
| 37 | MAT alpha | GCACGGAATATGGGACTACTTCG |
| 38 | HXT1_fw_q | GGCCGTCGTAACTGTTTGAT |
| 39 | HXT1_rv_q | AATTGGGGCCCAGGTAGTAG |
| 40 | HXT2_fw_q | TTGCCGAATCCTATCCTTTG |
| 41 | HXT2_rv_q | ACCAAACAGCCCATGAAGAC |
| 42 | HXT3_fw_q | GCTGACCTGCCTTCGAATAG |
| 43 | HXT3_rv_q | ACCGAAGGCAACCATAACAC |
| 44 | HXT4_fw_q | GTTGCTTTCGGTGGTTTTGT |
| 45 | HXT4_rv_q | AATGGCACAACCAATGTTGA |
| 46 | HXT5_fw_q | CAAGCGGTCCTTAGCAAGAG |
| 47 | HXT5_rv_q | ACCAACAGCCTGGAAAATTG |
| 48 | HXT6&7_fw_q | AAAGGTCCTTCAGCGTTTGA |
| 49 | HXT6&7_rv_q | GCACCCCATAGCAAACAAGT |

**Figure S1. Construction of Pdc negative strains.**

Figure S2. Bipartite strategy for gene deletion.

Figure S3.Reverse engineering strategy for *MTH1^81D^* integration into *MTH1* locus of E1 strain.

Figure S4. Adaptive evolution process of the Pdc negative strain E1.
