# Supplementary material for: Adaptive mutations in sugar metabolism restore growth on glucose in a pyruvate decarboxylase negative yeast strain
Source: Microb Cell Fact. 2015 Aug 8;14:116. doi: 10.1186/s12934-015-0305-6 (PMC4529725; doi:10.1186/s12934-015-0305-6)
Supplement: Supplementary file 3 — Additional file 3. Bioinformatics analysis of the mutations in Hxt2, Cit1 and Rpd3. [file 12934_2015_305_MOESM3_ESM.docx]

- Analysis of mutations in Hxt2 and their possible effects

The alignment results of Hxt2 from *S. cerevisiae* and other 201 homologous sequences from unicellular fungi revealed that the mutations G75R and W466* occurred at positions that are 100% and 93% conserved, respectively (Figure S5A). A homology model of Hxt2 was generated using the crystal structure of the human glucose transporter Glut1 with the aim of gaining insights into the function of G75 and W466 [[1](#_ENREF_1)]. As shown in Figure S5B, G75 is positioned in the interface between the N-and C-terminal domains, and W466 and the residues thereafter comprise one and a half trans-membrane alpha helices situated on the outside of the protein. A close-up of the region surrounding G75 revealed it’s positioned with the alpha carbon facing inwards in a bundle of four helices that form the substrate channel (Figure S5C). The mutation G75R at the 100% conserved residue could result in a very large change in side chain properties, since glycine has no side chain whereas arginine has the third largest side chain after tryptophan and tyrosine. Therefore, it is very likely that the G75R mutation completely disrupt the ability of this protein to mediate sugar transport due to steric hindrance. W466 is positioned at the middle of TM11, the second to last trans-membrane helix, which are structurally important and help in correctly positioning other helices with TM12 (Figure S5B&D). As shown in Figure S5D, the backbone of TM11 and the side chains of Y485 and Y489 on TM12 sterically push on residues Y333, F334, Y337 and I341 on TM7, causing a kink to form. The kink formed in TM7 brings it in contact with TM1 and together they represent the main constitutes of the extracellular gate of the substrate channel (Figure S5C). TM7 and TM1 form hydrogen-bonds in this region and mutations at theses residues are known to cause Glut1 deficiency syndrome in humans [[1](#_ENREF_1)]. However, it is hard to evaluate the effects of the mutation W466* due to the very large changes resulting from a 76 amino acid deletion. We speculated that the mutation W466* might relax the steric push on TM7, thus removing the kink and causing the substrate channel not form correctly, or may even cause complete misfolding of the protein.

- Analysis of mutations in Cit1 and their possible effects

The alignment results of Cit1 from *S. cerevisiae* and other 378 homologous sequences from fungi, animals and plants revealed that M84V, H175R and P176Q mutations occurred at positions that are 79%, 99% and 98% conserved, respectively (Figure S6A). A homology model of Cit1 was generated using the crystal structure of the chicken citrate synthase with the aim of gaining insights into the function of M84, H175, P176. As shown in Figure S6B, all three mutation are situated at the interface between the two polypeptides of the Cit1 homodimer. M84 is one of the highly conserved residues, which interact with the identical residue from the other polypeptide chain of the homodimer as shown in Figure S6C, and may serve structural and functionally important roles. Furthermore, R85 (R46 in chicken CS) hydrogen-bonds to the phosphate group of the substrate acetyl-CoA analogue, whereas R459 (R421 in chicken CS) to one carboxyl group of the substrate oxaloacetate. Therefore the M84V mutation might lead to a decreased Cit1 enzyme activity due to structural perturbations around the active sites, which in turn would lead to a decrease or loss of substrate binding affinity. As shown in Figure SD, H175 forms a water bridge with Q317 (97% conserved) from an opposing alpha helix in the same polypeptide chain, which likely helps increasing the structural rigidity. The mutation H175R may therefore lead to incorrect or inefficient protein folding or interfere with interactions between the two monomers. P176 likely serves a structural role initiating an alpha helix (Figure S6D), and the mutation P176Q may therefore cause the alpha helix not to form or to form later in the primary sequence, which may cause structural perturbation of several amino acids, including H175.

- Analysis of Rpd3 mutations and their possible effects

The alignment results of Rpd3 from *S. cerevisiae* and other 265 homologous sequences from unicellular and multicellular fungi, plants, animals and a range of bacteria revealed that the mutations F85I and A196V occurred at positions that are 97% and 98% conserved, respectively (Figure S7A). A homology model of Rpd3 was generated using the crystal structure of the human histone deacetylase 2 protein (PDB ID: 4LXZ) with the aim of gaining insights into the function of F85 and A196 [[2](#_ENREF_2)](Figure S7B). F85 is buried in between two alpha helices and a loop in the N-terminal domain of the protein (Figure S7B&C).While F85 is highly conserved, the residues surrounding it are less so. There is a trend that the inward-facing hydrophobic amino acids of this region, such as Y82, L86, V89 and V106, are among the more conserved at 98%, 94%, 58% and 85% respectively. In contrast, the more outward-facing hydrophilic residues, such as R88, E100 and S101, are less conserved at 35%, 39% and 35% respectively (Figure S7C). The data therefore indicate that F85 is important for the hydrophobic packing of this region and thus serves a structural role in the protein. A196 is part of an alpha helix that is situated at the interface between the N- and C-terminal domains. The helix is also immediately adjacent to the bound Ca^2+^ and Na^+^ ions (Figure S7D). All of the amino acids on this helix are highly conserved (D191 100%, G192 100%, V193 99%, E194 90%, E195 100%, A196 98%, F197 100% and Y198 96%). V167 (98% conversed) is directly interacting with A196 across the domain interface. The very high degree of conservation in this region of the protein structure is astounding, especially when one considers how evolutionarily diverse the set of sequences is. The residues are obviously very sensitive to mutations. It’s possible that the two extra methyl groups introduced by the A196V mutation stericly interfere with the side chain of V167, which may in turn cause a displacement of the conserved helix and result in decreased or abolished enzyme activity.


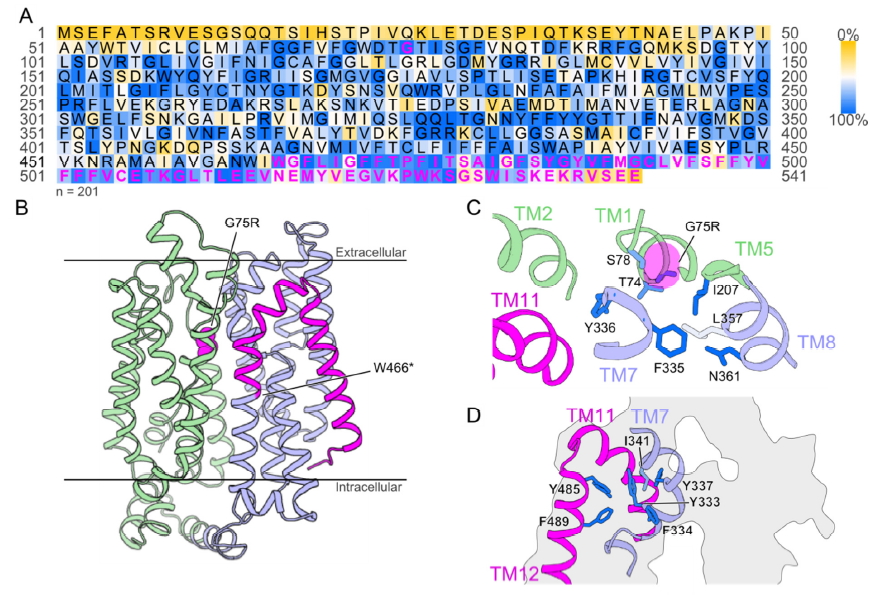


Figure S5. Mapping and analysis of Hxt2 mutations. A) Result of a multiple alignment using homologous sequences (n=201). The *S. cerevisiae* Hxt2 sequence is shown with colored conservation levels. Yellow indicates low conservation, white intermediate, and blue high. Magenta text indicates positions for the identified mutation and the amino acids missing due to the non-sense mutation. B) A cartoon representation of an Hxt2 homology model generated using a crystal structure of the human glucose transporter Glut1 (PDB ID: 4PYP). The N-terminal and C-terminal domain of the peptide are shown in pale green and purple, respectively. The global position of the mutation G75R is indicated by magenta sphere representation, and the mutation W466* by magenta coloring for the deleted protein sequence. Lines indicate the approximate boundaries of the phospholipid bilayer in which the protein performs its function. C) A close-up of the region surrounding G75R. D) A close-up of the region surrounding W466*. A protein cross-section is shown in grey with the substrate channel visible in white at the center of the protein. Amino acid sidechains are colored according their conservation using the same color as in A. The mutation is highlighted by a transparent magenta space-fill representation of the entire mutated amino acid. Hydrogen atoms in glycine are shown in order to visualize the residue position. The color-coded amino acid sequence was generated using custom software in Python and the structure was visualized using PyMol ([www.pymol.org](http://www.pymol.org)).


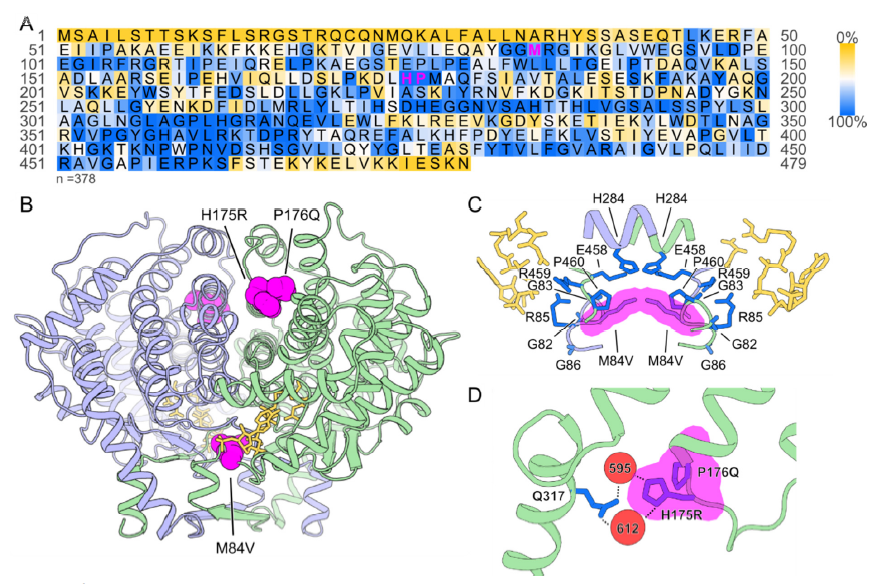


Figure S6. Mapping and analysis of Cit1 mutations. A) Result of a multiple alignment using homologous sequences (n=378). The *S. cerevisiae* Cit1 sequence is shown with colored conservation levels. Yellow indicates low conservation, white intermediate, and blue high. Magenta text indicates positions for non-synonymous mutations identified in this study. B) A cartoon representation of a Cit1 homology model generated using a crystal structure of chicken citrate synthase (PDB ID: 1AL6). The position of mutations is indicated by magenta sphere representations of the amino acids in the two polypeptides of Cit1 homodimer shown in pale purple and pale green respectively. The substrate oxaloacetate and the substrate analog N-hydroxyamido-CoA are shown in yellow. C) A close-up of the region surrounding M84V. Amino acid sidechains are colored according their conservation using the same color as in A. D) A close-up of the region surrounding H175R and P176Q. The mutation is highlighted by a transparent magenta space-fill representation of the entire mutated amino acid. Hydrogen atoms in glycine are shown in order to visualize the residue position. Water molecules are represented by red spheres and hydrogen bonds are indicated by dashed lines. The color-coded amino acid sequence was generated using custom software in Python and the structure was visualized using PyMol (www.pymol.org).


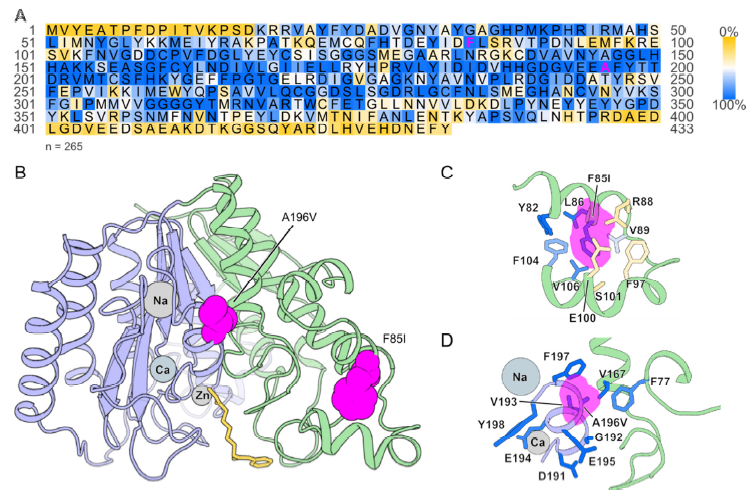


**Figure S7. Mapping and analysis of Rpd3 mutations. A) Result of a multiple alignment using homologous sequences (n=265). The *S. cerevisiae* Rpd3 sequence is shown with colored conservation levels. Yellow indicates low conservation, white intermediate, and blue high. Magenta text indicates positions for non-synonymous mutations identified in this study. B) A cartoon representation of an Hxt2 homologous model generated using a crystal structure of the human histone deacetylase 2 protein (PDB: 4LXZ). The N-terminal and C-terminal domain of the polypeptide are shown in pale green and pale purple respectively. The global position of mutations is indicated by magenta sphere representations of the amino acids. The enzyme inhibitor Vorinostat co-crystallized with the enzyme is shown in yellow. Metal ions in the structure are shown in grey colors (Na: sodium ion, Ca: calcium ion; Zn: zinc ion.). C) A close up of the region surrounding F85I. D)A close-up of the region surrounding A196V. Amino acid sidechains are colored according their conservation using the same color as in A. The mutation is highlighted by a transparent magenta space-fill representation of the entire mutated amino acid. Hydrogen atoms in glycine are shown in order to visualize the residue position. The color-coded amino acid sequence was generated using custom software in Python and the structure was visualized using PyMol (**[**www.pymol.org**](http://www.pymol.org)**).**

References

1. Deng, D., et al., *Crystal structure of the human glucose transporter GLUT1.* Nature, 2014. **510**(7503): p. 121-5.

2. Lauffer, B.E., et al., *Histone deacetylase (HDAC) inhibitor kinetic rate constants correlate with cellular histone acetylation but not transcription and cell viability.* J Biol Chem, 2013. **288**(37): p. 26926-43.
